# Inflated expectations: the strange craze for translational research on aging: Given existing confusion about the basic science of aging, why the high optimism in the private sector about the prospects of developing anti-aging treatments?

**DOI:** 10.1038/s44319-024-00226-2

**Published:** 2024-08-16

**Authors:** David Gems, Simon Okholm, Maёl Lemoine

**Affiliations:** 1 Institute of Healthy Ageing, and Research Department of Genetics, Evolution and Environment, University College London, London, WC1E 6BT UK; 2 CNRS, ImmunoConcEpT, UMR 5164, Univ. Bordeaux, Bordeaux, France

**Keywords:** History & Philosophy of Science, Molecular Biology of Disease, Pharmacology & Drug Discovery

## Abstract

The geroscience research program of the last decade has entailed a shift of focus in research on aging, away from understanding its underlying biology and towards translation into anti-aging treatments—a shift that is premature.

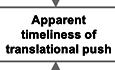

In November 2023, a large international conference took place at the Four Seasons Hotel in Riyadh, Saudi Arabia. The Global Healthspan Summit was hosted by the Hevolution Foundation, a new funding agency for research on aging (Khan et al, 2024). The product of a royal decree by the Prime Minister of Saudi Arabia, Crown Prince Muhammad bin Salman, Hevolution will provide around US$1B per year for research on aging, particularly the development of anti-aging treatments.

Representatives of numerous biotech companies came to the meeting, along with university scientists with startups on the side, and representatives of aging research institutes, many drawn by the promise of future funding. Over two days of talks and panel discussions, the imminent prospects for treatments for aging were talked up. The climax of the event was the announcement of the largest competition in history, the US$101M XPRIZE Healthspan, with funds from Hevolution and Peter Diamandis’ XPRIZE Foundation. This award is offered to the first research team to develop a treatment that rejuvenates muscle, cognition, and immunity function by a minimum of 10 years.

The emergence of Hevolution and the XPRIZE Healthspan is just the latest development in a remarkable phenomenon: a dramatic upsurge in activity in the private sector aimed at developing treatments for aging, fuelled by a heady optimism that the time is nigh (Ward, 2022). Other examples that involve massive funding to achieve practical outcomes in applied research on aging include the California Life Company (Calico), initiated by Google founder Larry Page, into which some US$2.5B has been invested; and Altos Labs, which in 2022 raised US$3B from investors, reportedly including Amazon founder Jeff Bezos.

Here we ask the question: what is the basis for this optimism? The last quarter of a century has seen a concerted effort by scientists to understand the fundamental biological mechanisms of aging, and much ground has been covered. How has this informed the recent upswell in commercial activity? We suggest that the latter is an anomaly arising in part from developments within the aging research field a decade ago that were, in some ways, counterproductive. These include the emergence of the so-called geroscience research agenda.

## Dazzling prospects for slowing human aging

What seems to have happened is the following. Advances in research on the biology of aging that culminated in the 1990s yielded startling implications. It seemed possible not only to understand the fundamental mechanisms of aging, but also to slow them down, at least in short-lived animal models. Consider the implications of the latter. In later life, human beings experience a vast panoply of degenerative changes—cardiovascular disease, chronic obstructive pulmonary disease, cancer, dementia, osteoarthritis, sarcopenia, and on and on—all wrought by the deteriorative process of senescence, that is, aging. It appeared that mechanisms existed that controlled the rate of appearance of the entire ugly gamut of diseases—mechanisms that could become potential targets for therapeutic intervention.

“It seemed possible not only to understand the fundamental mechanisms of aging, but also to slow them down, at least in short-lived animal models.”

Here, briefly, are some of the main reasons for optimism. Caloric restriction had long been known to postpone aging and extend life in rodents. Single-gene mutations were then found that increased lifespan in nematodes, fruit flies, and mice. This led to the identification of a growth-control system that also promotes aging, including growth hormone and mammalian target of rapamycin (mTOR) (Kenyon, 2010). Importantly, such mechanisms appeared to be active across much of the animal kingdom. Moreover, some animal species were found to display no increase in mortality with age, seemingly having evolved their own elixirs of immortality. Experimental evolution experiments proved able to substantially increase lifespan in fruit flies (Arnold and Rose, 2023). Growing evidence also seemed to support the view that the rate of aging is a function of molecular damage accumulation, including telomere shortening, caused particularly by reactive oxygen species, and the efficiency of the maintenance mechanisms that ameliorate such damage. This assumption was integrated with the evolutionary theory of aging into the disposable soma theory. Some analyses of human population aging concluded that there might be no upper ceiling to human lifespan (Vaupel, 2010). Together, such findings challenged earlier assumptions about the inexorable and unalterable nature of the aging process, and a fixed upper limit to human longevity. Heady times indeed.

These promising prospects led to the aging field becoming bigger and better, thanks to increased funding and the influx of many good scientists. As a result, standards of research grew more rigorous, including critical reassessments of earlier findings. Such careful research over the past two decades has, regrettably, undermined a number of the reasons for earlier optimism. Disappointingly, caloric restriction in rhesus monkeys proved not to have the same remarkable effects as those seen in rodents (Mattison et al, 2012), and even its effects in mice proved to depend on the strain used (Selman and Swindell, 2018). Growth hormone defects that extend lifespan in mice were found not to do so in humans (Aguiar-Oliveira and Bartke, 2019). Oxidative damage proved not to be a major, primary cause of aging (Perez et al, 2009; Shields et al, 2021), raising doubts about the importance of molecular damage more broadly, and about the disposable soma theory (Blagosklonny, 2007; Gems, 2022). Telomere shortening also proved to be of little causal relevance to aging (de Magalhães, 2024). The annual rise in worldwide maximum lifespan reached a ceiling at around the turn of the century, challenging the no-upper limit-claim (Dong et al, 2016). In the nematode *C. elegans*, where the largest increases in lifespan have been achieved (Kenyon, 2010), evidence emerged of self-destructive programs similar to those in spawning salmon, conferring a plasticity in aging not typical of higher animals (Kern et al, 2023). Overall, these results led to a dwindling, first in the prospects for understanding the mechanisms of aging in general terms, and then for the prospects of slowing it down in humans.

“Such careful research over the past two decades has, regrettably, undermined a number of the reasons for earlier optimism.”

## The rise of geroscience

The expansion of biogerontology in the 1990s was guided by a set of ideas about the causes of aging that constituted its guiding paradigm. Arguably, by the end of the 2000s that paradigm was crumbling, creating difficulties for the field and for the funding agencies that support it. With the dwindling likelihood that humans possess the plasticity in aging seen in shorter-lived animals, and the failure of existing theories of aging, how should one further pursue research?

Here two possible approaches may be envisaged. On the one hand, scientists could renew their efforts to develop an effective theoretical framework with the capacity to explain diverse phenomena of aging. This would be equivalent to the conceptual foundations of other, more mature scientific fields, such as chemistry with its periodic table of the elements and understanding of the nature of chemical bonds. Biogerontology currently lacks a foundation of this sort (Gems and de Magalhães, 2021). Such an approach involves hypothesizing, testing and reformulating, in the context of intensive discussion and debate.

On the other hand, research could focus on translating existing theoretical claims and experimental observations into therapeutic trials—preclinical or clinical. The pursuit of this strategy in the early 2010s marked the emergence of the geroscience agenda, particularly under the influence of the US National Institutes of Health and affiliated networks and interest groups (Okholm, 2024). A key idea was that it is sufficient to have a list of mechanisms that influence the rate of aging (Kennedy et al, 2014; López-Otín et al, 2013), to test interventions with potential generic effects on lifespan or healthspan. This approach approximates to a current mainstream in aging research, though it has not been universally embraced (Gems and de Magalhães, 2021).

“On the other hand, research could focus on translating existing theoretical claims and experimental observations into therapeutic trials—preclinical or clinical.”

Thus, in the face of a lack of fundamental assumptions about the nature of aging and how to study it, rather than going back to the drawing board to try to better understand aging, geroscience redirected research towards a practical, engineering-type strategy. Such an approach entails the beliefs that aging as a whole should be alterable, which sets the principal research objective; it should not be distinguished sharply from late-life diseases, which guides the choice of measuring tool for detecting efficacy; and its causes are too complex to explain in fundamental terms, which frees researchers from the necessity of fully explaining the results they obtain.

## Flying blind: premature translational research

The Global Healthspan Summit, which was attended by one of the authors (D.G.), embodied the geroscience mainstream par excellence. There was little discussion of the causes of aging, and some of the scientists present expressed the view that an understanding of aging in terms of basic principles is currently so unfeasible as to be unrealistic to pursue.

In the past, this strategy of prioritizing translational research in the absence of a good understanding of the basic science has sometimes proved successful, aided by brute force and serendipity. There are, however, also numerous instances where such trial-and-error approaches failed, sometimes involving investment of billions of dollars. The reader may pick their own examples. Regarding aging in particular, the field has a history of translational lost causes, such as antioxidant therapies and the debacle of resveratrol, including a lost US$720M investment by GlaxoSmithKline (Brenner, 2022).

Unquestionably, for translational research to yield useful, practical applications, at least some level of scientific understanding is required. At issue is judging when the time is ripe to move from basic to translational research, particularly where large investments of money and effort are involved. Of course, basic and translational research are rarely strictly successive but overlap in changing proportions, with a gradual shift in focus from the former to the latter. However, where big pushes to translation are concerned, these are often motivated by breakthroughs in basic science that reveal exciting and realistic opportunities for practical applications.

“At issue is judging when the time is ripe to move from basic to translational research, particularly where large investments of money and effort are involved.”

One evening in 1997, in a pub in Cambridge, UK, David Klenerman and Shankar Balasubramanian worked out the basic principles of high-throughput Illumina DNA sequencing, based on flow-cell chemistry. The realization of this revolutionary technology required a subsequent large-scale, 10-year-long translational research effort to develop the first next-generation sequencers. To have made such a translational push to develop high-throughput sequencing in 1987 would presumably have been premature; but with hindsight one can safely say that in 1997 the time was ripe for it. One may say the same of the launch of the US$10M XPRIZE in 1996 to whoever could develop the first commercially viable, reusable vehicle to carry passengers into space. This challenge, though difficult, was theoretically feasible thanks to the existing knowledge of physics and engineering—and the prize was won in 2004 by the developers of the SpaceShipOne spaceplane.

What, then, is the equivalent of that 1997 breakthrough in flow-cell chemistry that has ignited the imagination of the backers of the XPRIZE Healthspan, Calico, and Altos Labs? There seemed no clear answer to it at the Riyadh meeting. If so, this suggests that the recent rush to translation is premature. In turn, this begs the question: how is it that so many researchers and investors believe that the time is ripe for this big translational push? As a possible answer, we suggest the following tentative hypothesis.

**Figure 1:**
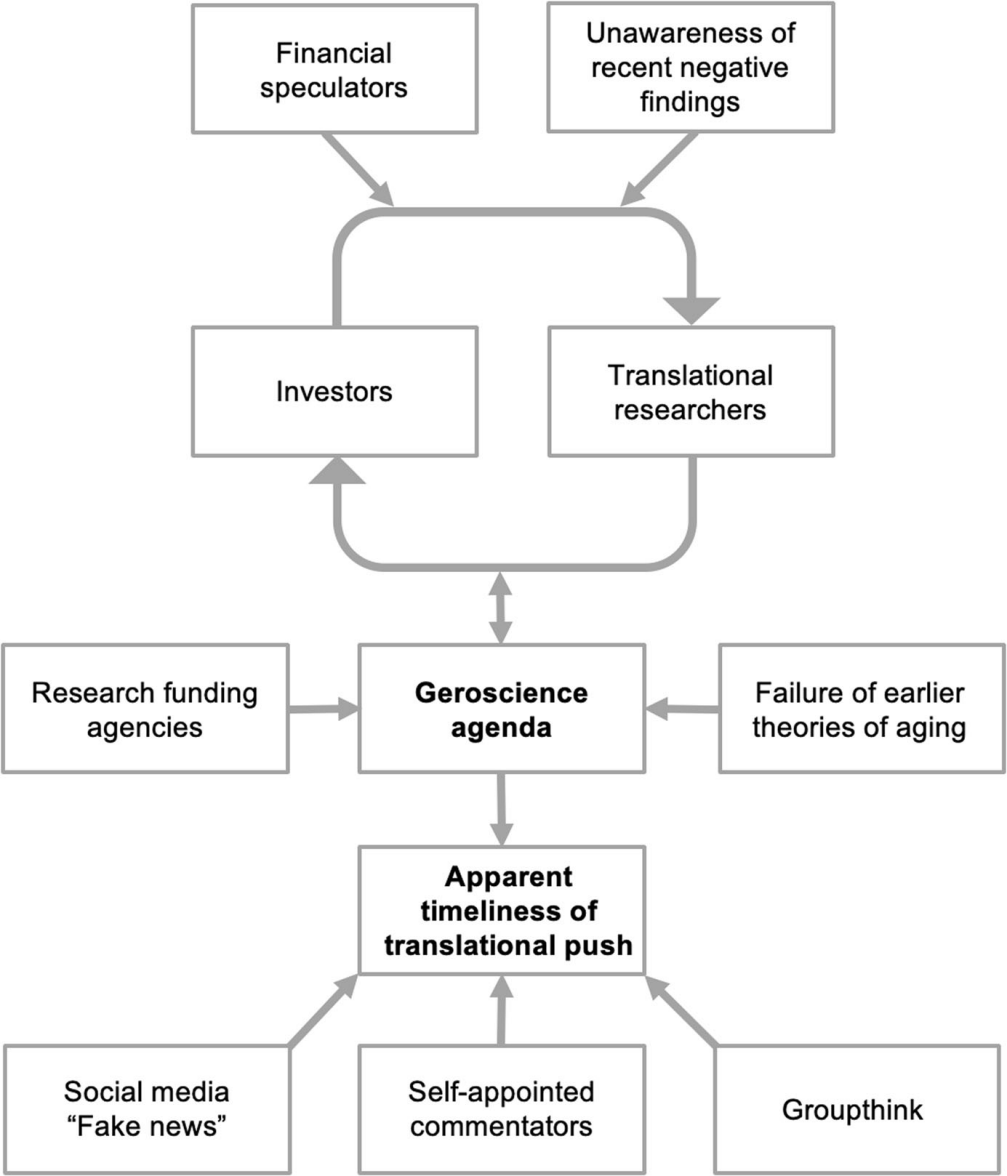
Hypothetical model for factors contributing to the premature push for translational research on aging. We suggest that an initial driver was the emergence in the early 2010s of the geroscience agenda in the wake of the crisis in theories of aging in the previous decade. This down-graded research aiming to understand aging in favor of applied research, and provided a research agenda for funding agencies. This in turn encouraged commercial translational research, and created a feedback loop of optimism between translational researchers and investors. This was further stoked by financial speculators, self-appointed commentators (mountebanks), and a lack of awareness of negative findings from basic research.

Several factors appear to have combined (Fig. 1). The first is a lack of realization in the commercial sector that key reasons for optimism that emerged in the 1990s by now appear less plausible. The second is a tendency for translational scientists to exaggerate the prospects of their research in order to attract investment. The resulting activity by investors can further increase optimism about research outcome, in a manner that creates a positive feedback loop (Fig. 1). Sociologists of science have referred to this as the sociology of expectations (Borup et al, 2006): a new regime of scientific research guided by visions, imaginings, strategies and agendas creating expectations, rather than exploration, recension and establishment of facts on which understanding becomes possible.

Other possible factors that stoke this amplification process include financial speculators, who may profit in the short-term by the increased value of biotech companies, whether or not actual translational benefits are eventually forthcoming; self-appointed commentators who make exaggerated claims about the prospects for treatments for aging; amplification of such non-expert views via the internet and social media; and, very likely, the reinforcing effect of groupthink (Janis, 1971). A final possible factor is the febrile hope that this field offers of the possibility of postponing inevitable death, which may be a further attractant for investors. In all, one might perceive in the present commercial translational research on aging the distinct lineaments of a bubble, with all the risks that that entails.

**Figure 2:**
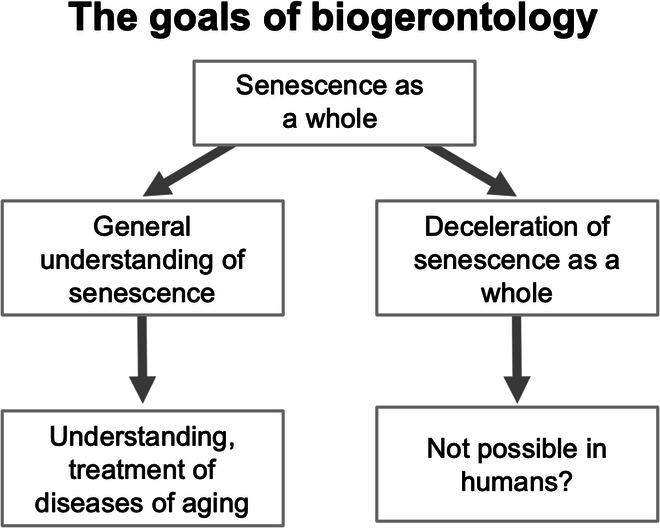
Two aspirations of research on the biology of aging. A defining feature of biogerontology is its attention to senescence (the aging process) as a whole, as opposed to its various consequences (e.g., late-life diseases) in piecemeal fashion. This includes *understanding* aging as a whole (left) and *treating* aging as a whole (right). Arguably, the prospects are improving for the former, but dwindling for the latter (at least, as far as human beings are concerned). But the former will be of incalculable value for understanding and preventing late-life diseases, individually or as multimorbidity.

The oddest thing about the translational geroscience approach, which was very much recapitulated at the Global Healthspan Summit, is its combination of pessimism about understanding aging, and optimism about translational research. Arguably, the inverse is more realistic (Fig. 2). Why should aging be impossible to understand? While there is currently no consensus with respect to general theories of aging, some emerging concepts show distinct promise, in particular—in our view—the programmatic theories, based on the evolutionary theory of aging (Arnold and Rose, 2023; Blagosklonny, 2007; de Magalhães and Church, 2005; Gems, 2022; Williams, 1957). Thus, there is at least some grounds for optimism about understanding aging in the years to come, leading to further insights into the causes of late-life diseases—and consequently a time that is truly ripe for major pushes in translational research. Billions of dollars have been spent on developing treatments for Alzheimer’s disease, with relatively little success. Arguably, this is because the process of aging that drives the development of this disease—that is, its *primary* causes—are not yet sufficiently understood.

“…there is at least some grounds for optimism about understanding aging in the years to come, leading to further insights into the causes of late-life diseases.…”

In the end, though, one should not lose sight of the fact that the Hevolution Foundation is a laudable initiative that will surely accelerate progress in the field of aging research to at least some degree. One can only hope that not too much of the funding that it proffers ends up disappearing into high risk or futile translational efforts, like so much water into desert sands.

## Supplementary information

Peer Review File
