# Supplementary material for: Inflated expectations: the strange craze for translational research on aging: Given existing confusion about the basic science of aging, why the high optimism in the private sector about the prospects of developing anti-aging treatments?
Source: EMBO Rep. 2024 Aug 16;25(9):3748–52. doi: 10.1038/s44319-024-00226-2 (PMC11387832; doi:10.1038/s44319-024-00226-2)
Supplement: Supplementary file 1 — Peer Review File [file 44319_2024_226_MOESM1_ESM.pdf]

EMBO reports

Inflated expectations: the strange craze for translational research on aging

David Gems, Simon Okholm, and Maël Lemoine

Corresponding author(s): David Gems (david.gems@ucl.ac.uk)

Review Timeline:

	Submission Date:	21st May 24
	Accepted:	26th Jul 24

Editor: Holger Breithaupt

Transaction Report: This manuscript was reviewed editorially and by an external expert.
